# Data in support of the bone analysis of NOD–SCID mice treated with zoledronic acid and prednisolone

**DOI:** 10.1016/j.dib.2016.04.041

**Published:** 2016-04-23

**Authors:** Naoko Hori, Takahiro Abe, Tsuyoshi Sato, Shoichiro Kokabu, Yumiko Shimamura, Tomoya Sato, Tetsuya Yoda

**Affiliations:** a Department of Oral and Maxillofacial Surgery, Japan; b Department of Plastic and Reconstructive Surgery, Faculty of Medicine, Saitama Medical University, Japan; c Department of Oral and Maxillofacial Surgery, The University of Tokyo, Japan; d Department of Molecular Signaling & Biochemistry, Kyushu Dental College, Japan

**Keywords:** Zoledronic acid, NOD–SCID mice, Medication-related osteonecrosis of the jaw

## Abstract

This paper reports data on the bone, specifically the tibia and mandible, of nonobese diabetic mice with severe combined immunodeficiency disease (NOD–SCID mice) treated with zoledronic acid (ZA) and prednisolone (PSL). The data described here are related to the research article titled “Zoledronic acid basically increases circulating soluble RANKL level in mice, and in glucocorticoid-administrated mice, more increases lymphocytes derived sRANKL by bacterial endotoxic stimuli” [1]. The present data and the NOD–SCID mice experiments described contain insights into the role of bone-remodeling factors induced by ZA treatment.

## 1 **Specifications table**

**Table:** 

Subject area	Biology
More specific subject area	Bone metabolism
Type of data	Figures
How data were acquired	Microscope, bone histomorphometry
Data format	Raw, images, analyzed
Experimental factors	Implantation of prednisolone pellets in NOD–SCID mice
Experimental features	Quantitative and qualitative analysis of bone structure
Data source location	Bunkyo-ku, Tokyo, Japan
Data accessibility	The data are supplied with this article

## 2 **Value of the data**

• The methodology provided can be used to evaluate the relationship between the immune response and ZA in bone metabolism.
• These data are valuable to researchers benchmarking bone morphological changes according to different medications.
• This approach would be useful for researchers studying the pathophysiology of medication-related osteonecrosis of the jaw.

## 3 **Data**

These data provide supporting information related to a comparison of the bone analysis of NOD–SCID mice and C57BL/6JJc1 mice and circulating bone-remodeling factors [1]. Bone analysis of NOD–SCID mice revealed a greater reduction in osteoblastogenesis with ZA treatment than without treatment. To determine whether ZA treatment affects the bone volume and structure of the NOD–SCID mouse mandible, we used microfocus X-ray computed tomography (μCT) to assess the mandibular microarchitecture, specifically, the bone volume/tissue volume and trabecular thickness.

## 4 Experimental design, materials and methods

### 4.1 Experimental protocol

**Fig. 1:**
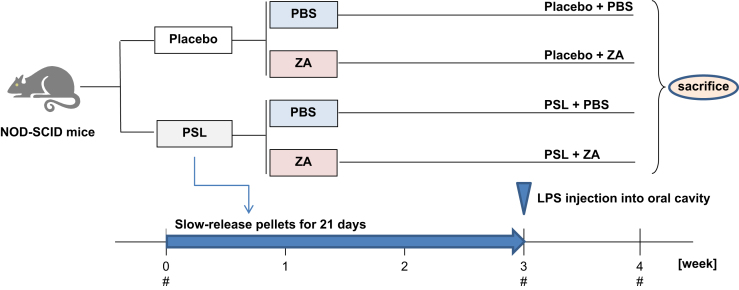
Schematic of the animal experimental protocol. PBS, phosphate-buffered saline; PSL, prednisolone; ZA, zoledronic acid; #, blood sampling from tail vein.

**Fig. 2:**
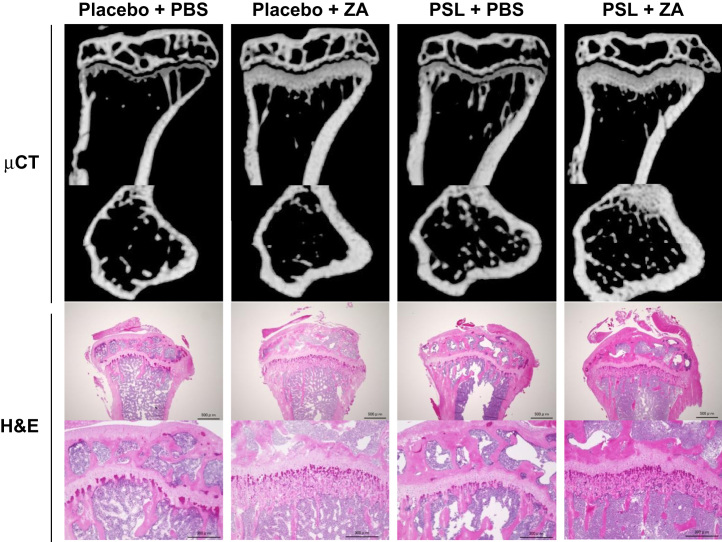
Representative images of the proximal region of the tibia in NOD–SCID mice.

**Fig. 3:**
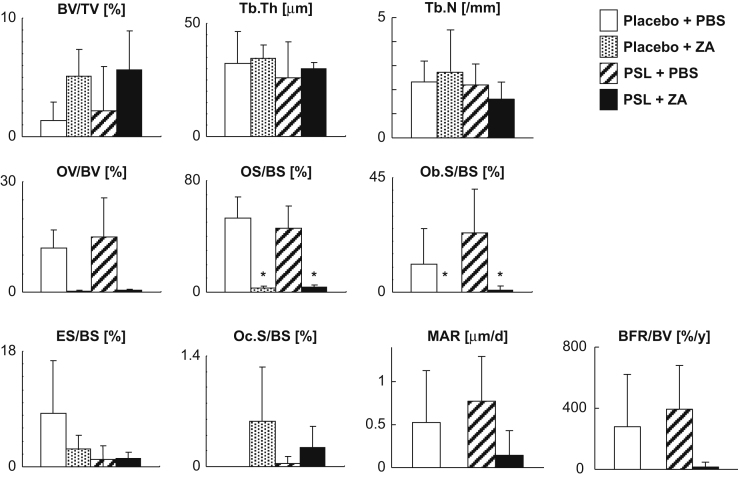
Bone histomorphometric analysis of the tibia in NOD–SCID mice. BV/TV, bone volume/tissue volume; Tb.Th, trabecular thickness; Tb.N, trabecular number; OV/BV, osteoid volume/bone volume; OS/BS, osteoid surface/bone surface; Ob.S/BS, osteoblast surface/bone surface; ES/BS, eroded surface/bone surface; Oc.S/BS, osteoclast surface/bone surface; MAR, mineral apposition rate; BFR/BV, bone formation rate/bone volume. *p<0.05 Compared with placebo+PBS group. *n*=4 For each treatment group.

**Fig. 4:**
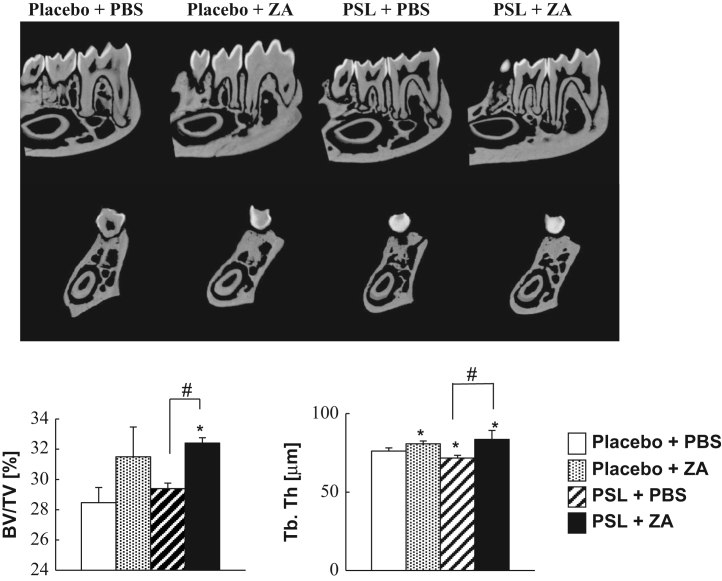
μCT analysis of the mandible of NOD–SCID mice. BV/TV (%), bone volume/tissue volume; Tb.Th (μm), trabecular thickness. *p<0.05 Compared with placebo+PBS group. ^#^*p*<0.05 Compared with the PSL+PBS and PSL+ZA groups. *n*=3 For each treatment group.

Animal experiments were conducted by reference to the ARRIVE Guidelines for Reporting Animal Research [2] and have been described in detail previously [1]. Male NOD.CB17-Prkdc^scid^/J, so-called NOD–SCID, mice (Charles River Japan, Yokohama, Japan) were obtained at 8 weeks of age. Fig. 1 shows a schematic of the animal experimental protocol. For glucocorticoid and bisphosphonate (BP) administration, slow-release pellets (Innovative Research of America, Sarasota, FL) of placebo or PSL 3.5 mg/kg/day were implanted into the lateral side of the neck of the mice, according to manufacturer׳s protocol. BP as zoledronic acid (ZA) was provided by Novartis Pharma AG (Basel, Switzerland). We treated the NOD–SCID mice with once-weekly subcutaneous injections of 100 μg/kg ZA or with phosphate-buffered saline (PBS) alone. PSL and placebo pellets implanted into the NOD–SCID mice were released over 21 days, and the weekly injections continued throughout the 21-day release period (Fig. 2, Fig. 3, Fig. 4).

### 4.2 Microfocus X-ray computed tomography

Microfocus X-ray computed tomography (μCT) (ELE SCAN; Nittetsu Elex Co. Ltd., Tokyo, Japan), following the manufacturer׳s protocol, was used to obtain cross-sectional views of the secondary spongiosa in the distal tibiae metaphysis at about 0.28 mm distal to the growth plate, with energy 67.0 kV, current 100 μA, and slice thickness 13.09 μm (*n*=4 animals per group). The mandible underwent microarchitectural analysis with μCT. The scanning conditions of the mandible were a voxel size of 10 μm with an X-ray tube voltage of 67 kV and intensity current of 101 μA. The microarchitectural properties of the mandible specimens were evaluated within a conforming volume of interest, which was defined as the bone area between the first molar and second molar.

### 4.3 Bone histomorphometry

The bony specimens were fixed, left undecalcified, and embedded in ethanol (70–80%), which was 25-times the volume of the sample, based on the method of a previous study [1]. For calcein labeling, 4 days and 1 day before the sacrifice of the NOD–SCID mice, four mice in each group were given an intraperitoneal injection of calcein (16 mg/kg; Dojindo, Kumamoto, Japan) for double labeling. A computer and digitizer tablet (Histometry RT Camera; System Supply Co., Ltd., Nagano, Japan) were used for histomorphometric examination, which was conducted by the Niigata Bone Science Institute (Niigata, Japan). Usage of nomenclature, symbols, and units followed the recommendations of the Nomenclature Committee of the American Society for Bone and Mineral Research [3].

### 4.4 Statistical analysis

Each result is the mean±SEM of three replicate measurements. Data were compared using one-way analysis of variance (ANOVA) or Student׳s *t*-test and the threshold for statistical significance was set at *p*<0.05.
